# Abnormal Prothrombin (PIVKA-II) Expression in Canine Tissues as an Indicator of Anticoagulant Poisoning

**DOI:** 10.3390/ani11092612

**Published:** 2021-09-06

**Authors:** Lorella Maniscalco, Katia Varello, Simona Zoppi, Giuseppina Abbamonte, Marta Ferrero, Elena Torres, Federica Ostorero, Francesca Rossi, Elena Bozzetta

**Affiliations:** Istituto Zooprofilattico Sperimentale del Piemonte, Liguria e Valle d’Aosta, 10154 Torino, Italy; lorella.maniscalco@izsto.it (L.M.); simona.zoppi@izsto.it (S.Z.); giseppina.abbamonte@izsto.it (G.A.); marta.ferrero@izsto.it (M.F.); elena.torres@izsto.it (E.T.); federica.ostorero@izsto.it (F.O.); francesca.rossi@izsto.it (F.R.); elena.bozzetta@izsto.it (E.B.)

**Keywords:** PIVKA-II, anticoagulants, poisoning, canine

## Abstract

**Simple Summary:**

PIVKA-II is an aberrant form of vitamin K that has been demonstrated to be increased in human coagulation disorders and in some neoplastic diseases. In veterinary medicine, PIVKA-II levels have been demonstrated to be useful for distinguishing anticoagulant poisoning from other coagulopathies. In forensic pathology, there is the need to distinguish malicious poisoning from other causes of death and, in some cases, identifying poisoned dogs from dogs that died as a result of other coagulative disorders can be challenging. This study evaluated the usefulness of the immunohistochemical expression of PIVKA-II in hepatic and renal tissues in order to identify patients with coagulative disorders due to clinical condition or the ingestion of anticoagulants substances.

**Abstract:**

PIVKA-II is an aberrant form of vitamin K that has been demonstrated to be increased in human coagulation disorders and in some neoplastic diseases. In veterinary medicine, PIVKA-II levels have been demonstrated to be useful for distinguishing anticoagulant poisoning from other coagulopathies. In forensic pathology, there is the need to distinguish malicious poisoning from other causes of death and, in some cases, identifying poisoned dogs from dogs that died as a result of other coagulative disorders can be challenging. In this study, dogs that suddenly died underwent necropsy, histological examination, and toxicological analysis to establish cause of death. PIVKA-II immunohistochemical expression was evaluated on hepatic and renal tissues, and on neoplastic lesions when present. A total of 61 dogs were analyzed and anticoagulant substances were identified in 16 of the 61. Immunolabelling for PIVKA-II was observed in 27 of 61 cases in the liver and in 24 of 61 cases in the kidneys. Among the poisoned dogs, the PIVKA-II expression was present in the liver in 15 of 16 cases and in the kidneys in 16 of 16. Neoplastic lesions represented mainly by haemangiosarcomas were negative. This study highlights how the immunohistochemical expression of PIVKA-II in hepatic and renal tissues can be useful to identify patients with coagulative disorders due to clinical condition or the ingestion of anticoagulants substances.

## 1. Introduction

Vitamin K, discovered by Dane Henrik Dam in 1930, is an essential cofactor in the carboxylation of hepatic coagulation factors (F), i.e., prothrombin (FII), FVII, FIX, and FX; proteins C, S, Z, and M; and several extrahepatic vitamin-K-dependent proteins [1]. Vitamin K is an essential cofactor for the γ-carboxylation of key glutamic acid residues in blood clotting proteins by vitamin K-dependent carboxylase [2]. Acute vitamin K deficiency is classically characterized by deranged coagulation because of insufficiency of vitamin K-dependent coagulation factors, but it can also be induced by action of vitamin K antagonists such as warfarin, used therapeutically as oral anticoagulants.

Coumarin anticoagulants are considered as antivitamin K [3]. During catalysis, vitamin K is converted from the active form to vitamin K 2,3-epoxide, which must be recycled to the active form by vitamin K epoxide reductase (VKOR) to maintain the coagulation cycle. 4-Hydroxycoumarins antagonize VKOR, preventing vitamin K recycling and resulting in an accumulation of an abnormal form of coagulation protein called des-γ-carboxyprothrombin (DCP) or proteins induced by vitamin K antagonism (PIVKA-II) [4].

PIVKA-II concentrations increase when hepatic vitamin K stores are sufficiently low in order to prevent the effective γ-carboxylation of factor II when the vitamin K epoxide reductase activity is reduced or when the γ-glutamyl carboxylase GGCX activity is insufficient. Elevated PIVKA-II precedes any subsequent change in PT [5].

PIVKA-II is also considered a retrospective indicator of vitamin K status [2]. All of these applications have been extensively investigated in human medicine where PIVKA-II serum concentrations are demonstrated to be increased in coagulation disorders and in some neoplastic disease such as in hepatocellular carcinoma [6,7,8,9] and pancreatic cancers [10]. During the process of malignant transformation in hepatocytes, PIVKA-II is produced as a defect in posttranslational carboxylation [11]. In this process, PIVKA-II loses its normal prothrombin function but may take on an important role promoting malignant proliferation in hepatocellular carcinoma [11,12]. In canine species, in addition to the above mentioned causes of vitamin K deficiency, there are also superwarfarin-type vitamin K antagonists [13] that are widely employed as rodenticides, which are among the most common causes of poisoning in dogs worldwide [14].

The Istituti Zooprofilattici Sperimentali (IIZZSS) laboratories are part of the state veterinary laboratories that fall under the umbrella of the Ministry of Health, and are designated as the local reference laboratories for monitoring and conducting toxicological analyses, in close collaboration with judicial authorities. According to the provisions of Law No. 189 of 2004 and the Ministerial Order of 18 December 2008, and following amendments, the use of poisons and the killing of animals are illegal and are defined as a crime. In veterinary medicine, PIVKA-II was first investigated by Mount and colleagues in 2003, who measured PIVKA-II levels in plasma, demonstrating that this is a diagnostically useful method for distinguishing anticoagulant poisoning from other coagulopathies [15].

Although in human medicine the PIVKA-II expression in tissues has been evaluated for its importance as a prognosticator in cancer, to the best knowledge of the authors, PIVKA-II has not been previously investigated in the veterinary field, so the aim of this work is to consider the PIVKA-II expression in canine tissues as a useful diagnostic tool to distinguish whether the cause of death was as a result of anticoagulant poisoning or some other conditions.

## 2. Materials and Methods

Cadavers of dogs suspected to be poisoned, and thus brought to the Diagnostic Service of Istituto Zooprofilattico Sperimentale del Piemonte, Liguria e Valle d’Aosta between 2018 and 2020 for establishing the cause of death, were included in the present study. Gross examination of the whole body and organs was performed, followed by histology of the selected organs (lungs, kidneys, liver, spleen, and gastrointestinal tract, including grossly lesioned organs) for all of the animals was carried out. Toxicological screening on the target organs (kidney and liver) and gastric contents of all of the dead animals was performed, as well as immunohistochemistry on the organs, using an antibody against PIVKA II.

### 2.1. Toxicological Analysis

Semi quantitative analytical methods were applied for the detection of >50 toxic substances in the samples collected from suspected poisoned animals during the necropsy or inspection exam, respectively. A critical step in this kind of analysis is the development of fast multi-residue methods for the determination of multiple different molecular classes in heterogeneous matrixes, for example, target organs (kidney and liver), gastric contents, and baits. To speed up the treatment, a two-step extraction technique was performed based on the QuEChERS approach, followed by a clean-up step with solid-phase dispersion extraction (dSPE). After concentration at 50 °C in a nitrogen stream, purified extracts were used for the instrumental analysis.

For the LC−MS analysis, 1 mL of purified extract was evaporated to dryness at 50 °C in a nitrogen stream, dissolved with 0.4 mL of a methanol/water (1/1) mixture, and transferred to a vial. For the GC–MS analysis, 1 mL of purified extract was evaporated to dryness, dissolved with 0.1 mL methanol, and transferred to a vial.

Analytical chemistry was performed to determine the presence of carbamates, organophosphates, organochlorines, pyrethroids and rodenticides, anticoagulants, strychnine, alpha-chloralose, and metaldehyde.

As a result of the differences in the physicochemical properties of the poisons, liquid chromatography−mass spectrometry (LC−MS) and gas chromatography–mass spectrometry (GC–MS) were performed to detect the substances. One aliquot of purified extract was injected in a liquid-chromatograph Agilent 1100 (Agilent Technologies, St. Clara, CA, USA) coupled with a tandem mass spectrometer system API4000 (Sciex, Framingham, MA, USA), operating in ESI positive and negative MRM mode, and at least two transitions for each analyte were acquired. This instrumental technique allowed for detect anticoagulants, alpha-chloralose, carbamates, strychnine, chlorpyriphos methyl, coumaphos, methamidophos, mevinphos, and parathion methyl. Another aliquot of extract was injected in a gas-chromatograph Agilent 6090 N coupled with a single quadrupole mass spectrometer Agilent 5975 system (Agilent Technologies, St. Clara, CA, USA), operating in full scan mode with EI ionization, in order to detect the more volatile compounds, namely: alpha-endosulfan, beta-endosulfan, endosulfan-sulfate, lindan, metaldehyde, piperonyl butoxide, ethylene glycol, cypermethrin, deltamethrin, and permethrin I and II.

The analyses were performed as described elsewhere [16].

### 2.2. Histological Examination

Samples from the selected organs were fixed in 4% neutral buffered formalin, and were paraffin embedded, sectioned at 4 μm, and stained with hematoxylin and eosin (HE). To establish the cause of death, histological samples were examined by two independent pathologists (L.M. and K.V.).

### 2.3. Immunohistochemistry

Immunohistochemical staining against PIVKA-II was performed. Briefly, paraffin sections were deparaffinized and rehydrated through a graded alcohol series. The sections were then placed in a 10 mmol/L citrate buffer (pH 6.0), heated at 95 °C 30 min, and then cooled in the box at room temperature (24 °C) for 60 min before being immersed for 5 min in Peroxidazed 1^®^ Blocking Reagent in order (BioCare Medical, Pacheco, CA, USA) to block the endogenous peroxidase. The primary monoclonal antibody, anti-PIVKA-II (Rabbit polyclonal antibody against PIVKA-II; 1:2000 dilution, synthesized by commission by Diatheva Srl, Cartoceto, Italy), was applied to the sections and was incubated 2 h at 4 °C in a moist chamber. The specificity of the antibody was assessed using Western blot analysis (Figure S1). The slides were labeled with DAKO anti-mouse Envision+ System (Agilent Dako, Santa Clara, CA, USA). Chromogenic fixation was carried out by immersing the sections in 0.05% *w*/*v* 3,3-diamino-benzidine tetrahydrochloride (DAB) at room temperature (24 °C) for 5 min. The sections were then counterstained with Mayer’s hematoxylin. The negative control sections were incubated with the immunoglobulin fraction of the mouse non-immune serum, instead of the primary antibody. The immunostained sections were examined under a light microscope and were evaluated by two independent observers who were unaware of the clinical and histological diagnoses.

### 2.4. Statistical Analysis

The IHC results and clinicopathologic findings were grouped into contingency tables and were analyzed using Fisher’s exact test or χ^2^ test. Data were analyzed with MedCalc Statistical Software v.13.3 (MedCalc Software bvba, Ostend, Belgium); *p* < 0.05 was considered statistically significant.

## 3. Results

A total of 61 dogs were analyzed. Among them, 27 (44.26%) were female and 34 were male (55.74%). Exact data regarding age of the animals were not available for all of the subjects, but the group was represented by adult subjects (more than 1 year old). The results are summarized in Table 1.

### 3.1. Toxicological Results

The toxicological results revealed that 20 of 61 (32.79%) dogs had been poisoned with different types of substances, and among them anticoagulant substances were found in 16 of 20 (26.23%) dogs (results summarized in Table 2).

The anticoagulants identified were mainly brodifacoum and bromadiolone or a mixture of these substances, while in one case, coumatetralyl was identified. Other toxic substances were methomyl (insecticide), embutramide (opioid analgesic), strychnine, and alphachloralose (non-anticoagulant rodenticide with narcotic effects).

### 3.2. Necroscopical and Histological Evaluation

Gross lesions suggestive of a coagulative disorder were observed in 24 cases (39.34%), of which 16 (26.23%) showed positive results for anticoagulant substances. In eight dogs (13.11%), the cause of death was assessed to be associated with disseminated intravascular coagulation due to massive pulmonary strongylosis (1/8) or gastric dilation (1/8), or was correlated with sepsis (6/8). In four dogs (6.6%), macro- and micro-scopic hepatic degeneration was highlighted, while in three (4.92%) cases severe renal tubular degeneration and interstitial fibrosis was observed

In 12 dogs (19.67%), during examination of the abdominal cavity, hemorrhagic effusion was observed and splenic neoplasia identified as hemangiosarcoma by histology was diagnosed.

### 3.3. Immunohistochemistry

**Figure 1:**
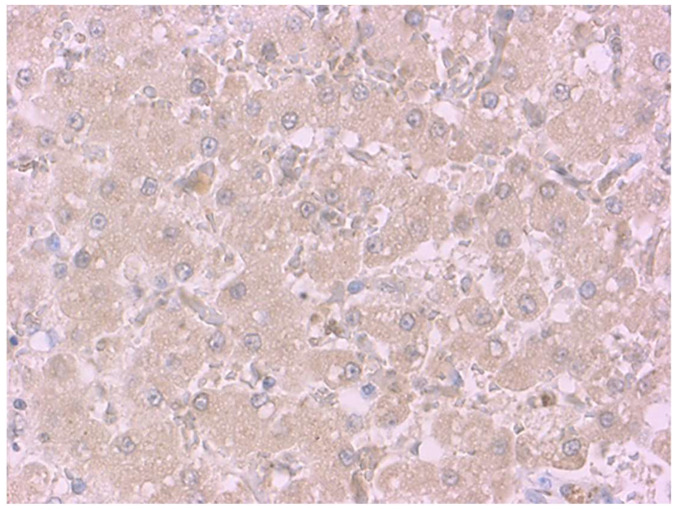
Liver of a dog after PIVKA-II immunostaining. Positive diffuse cytoplasmic immunoreactivity within hepatocytes. PIVKA-II immunohistochemistry, Mayer’s hematoxylin counterstain, and 40× objectives.

Immunohistochemistry was performed in the renal and hepatic tissues of all of the subjects; moreover, in cases in which haemangiosarcoma was present, PIVKA-II was also investigated in the neoplastic tissue. Immunolabelling for PIVKA-II was observed in 27 of 61 (44.26%) cases in the liver and in 24 of 61 (39.34%) cases in the kidney (Table 3). Positivity in the hepatic tissue was uniformly distributed among hepatocytes with cytoplasmic immunolabeling (Figure 1), and the intensity of staining was variable, from weak to strong.

**Figure 2:**
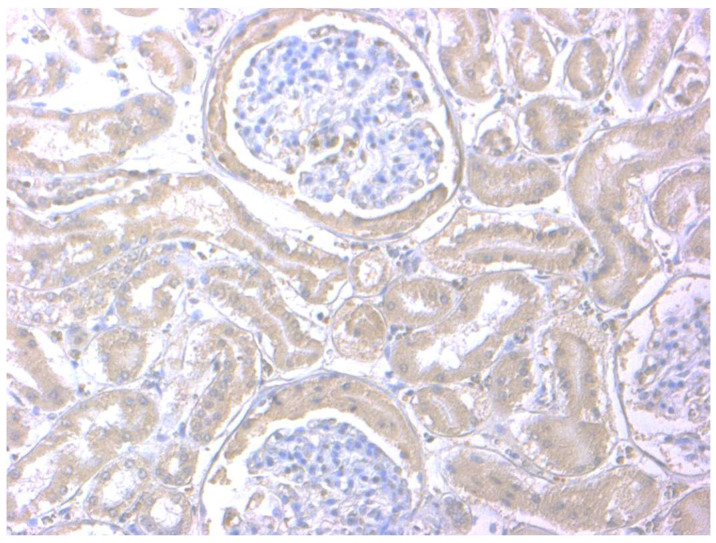
Kidney of a dog after PIVKA-II immunostaining. Positive cytoplasmic immunoreactivity within the renal tubules and Bowman’s capsule. PIVKA-II immunohistochemistry, Mayer’s hematoxylin counterstain, and 20× objectives.

In the kidneys, positivity was observed mainly in the renal tubules, and in two cases also in the Bowman’s capsule (Figure 2).

**Figure 3:**
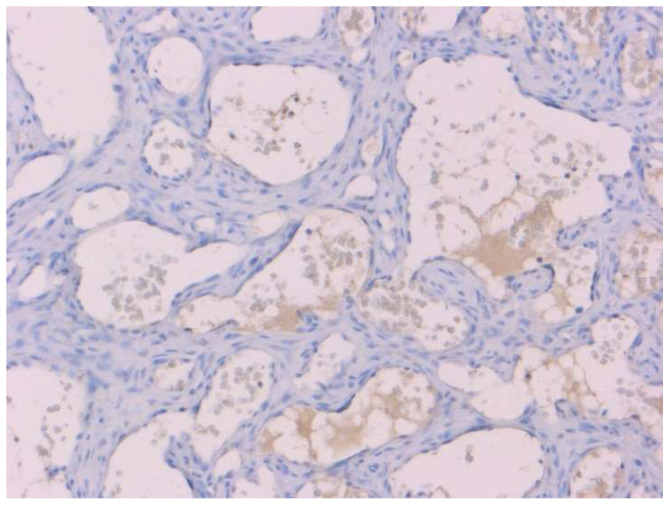
Hemangiosarcoma of a dog after PIVKA-II immunostaining. Negative in the neoplastic cells. PIVKA-II immunohistochemistry, Mayer’s hematoxylin counterstain, and 20× objectives.

When haemangiosarcoma was diagnosed, immunohistochemistry against PIVKA-II was carried out with negative results for all of the cases (Figure 3).

The results regarding positivity distribution in the dogs that tested positive and negative for anticoagulant substances are listed in Table 1. Statistical association between positivity in both the liver and kidney in dogs in which anticoagulant substances were isolated was found (Fisher’s Exact test *p* ≤ 0.005).

Positivity for PIVKA-II was also observed in some cases that presented negative results anticoagulants substances (45 cases)—12 of 45 cases in the liver and 8 of 45 cases in kidneys. Eight dogs that tested negative for anticoagulants had positive immunolabeling for PIVKA-II in both the kidneys and liver; in those cases, cause of death was assessed as due to intravascular disseminated coagulation. In the remaining four negative cases, in which only hepatic positivity was revealed, a diffuse hepatic microvacuolar degeneration was observed.

## 4. Discussion

To the best of the authors’ knowledge, this is the first study to examine PIVKA-II expression in canine tissues. PIVKA-II is an abnormal prothrombin generated by liver cells under conditions of reduced vitamin K or in the presence of a vitamin K antagonist [13,17]. Since the 1990′s, PIVKA-II has been widely used as a human marker of hepatocellular carcinoma, demonstrating that an aberrant form of vitamin K can be produced by a lack of vitamin K induced by anticoagulant administration, coagulation disorders, and neoplastic modification of the hepatic tissue [17,18,19,20]. This aberrant prothrombin is used as an indicator of blood coagulability in human medicine, and the same role was demonstrated in canine specie by Mount and colleagues [15]. In their study, they demonstrated that a PIVKA-II test on canine plasma samples was diagnostically useful for distinguishing anticoagulant poisoning from other coagulopathies [15].

Recent studies have indicated that, in <4% of animals, cause of death was poisoning, sometimes via the simultaneous use of harmful and toxic substances with the intention to maximize pain in the victim (Di Blasio et al., 2020). More often, pet owners who found their animals dead requested forensic necropsy suspecting a malicious poisoning. It is important to rapidly and accurately screen suspected deadly cases of rodenticide poisoning in order to act and prosecute perpetrators properly.

In some cases, toxicological substances can easily be evaluated by chemical examination of the gastric content and other tissues, but sometimes, in very acute or chronic cases, it can be challenging. Histological exams could contribute to discovering whether fatal cases are related or unrelated to anticoagulant poisoning, particularly where gross lesions are mixed or confused by concurrent pathologies. The use of another method could be of some help to better improve the accuracy when establishing the cause of death.

Based on what has been described by Mount and colleagues, who investigated the PIVKA-II expression in the plasma of dogs, we investigated the expression of the aberrant form of vitamin K (PIVKA-II) in the tissues of animals in which poisoning was suspected to be de cause of death, observing that it could be expressed in the hepatic and renal tissue of dogs that died as a result of anticoagulant poisoning (100% of the cases), but also in some subjects that died from coagulative disorders or having hepatic degeneration.

It is not surprising that in cases of hepatic degeneration, PIVKA-II is weakly expressed due to liver dysfunction induced coagulative disorders [21]. This result appears to be different from that observed in some studies carried out on human hepatic carcinomas tissues, in which PIVKA-II seemed to be strongly expressed in the neoplastic cells and was negative in the adjacent cirrhotic hepatic tissue [17,22]. On the contrary, our results are in agreement with that observed in a previously cited study carried out on canine sera, which demonstrated that the PIVKA-II test can also be increased by deficiencies in factors II, VII, and X of heredity and acquired coagulopathies [15], and with what was described in a study carried on feline sera, which also observed a prolonged PIVKA clotting time in patients with hepatic pathologies [23]. As in those studies a PIVKA-II cut-off value in serum was assessed to be indicative of anticoagulant poisoning, it could be possible that in our cohort of cases, a correlation with poisoning would have been found if a quantitative method of evaluation had been adopted. In addition, we found the PIVKA-II expression in hepatic or renal tissues in the case of DIC. This is not surprising, because the PIVKA test is widely used to in human medicine as a screening test to differentiate whether or not vitamin K deficiency is present in various clinical cases, such as patients with liver diseases, disseminated intravascular coagulation, and other clinical conditions [20,24,25,26,27]. The main limitation of this study is the lack of sera collected before death in the animals of this study, and consequently the lack of correlation between the PIVKA-II level in the serum and biochemical serum enzyme levels of the main indicators of both hepatic and renal dysfunction; thus, further intra vitam studies are necessary to better understand the role of PIVKA-II. In addition, further studies are required to evaluate if the PIVKA-II test can also be used on the plasma collected during necroscopical examination in canine species, as described by Rutty and colleagues in 2003 for autopsy investigation in human forensic pathology [27].

Moreover, the response to anticoagulants is quite variable, resulting in different commercially available substance classes, dose-related toxicities, animal species, or repeated exposure, including liver bioaccumulation. Therefore, further studies are required for establishing if PIVKA-II could potentially be employed as an effective predictive biomarker in the diagnosis of rodenticide poisoning in dogs as well as in other animal species.

## 5. Conclusions

This study demonstrates the PIVKA-II expression in the canine tissue of animals with coagulopathies induced by anticoagulant substances with a high sensitivity but a low specificity, because it is also present in tissues of dogs with pathologies induced by coagulative disorders.

## 6. Patents

This section is not mandatory but may be added if there are patents resulting from the work reported in this manuscript.

## Figures and Tables

**Table 1:** Characteristics (age and gender), toxicological results, cause of death, and immunohistochemical result of each case. DIC (disseminated intravascular coagulation); + (positive); − (negative).

	Age (Years)				PIVKA-II IHC Results	
Case n.		Gender	Toxicology	Cause of Death	Liver	Kidney
1	4	Male	Brodifacum	Intoxication	+	+
2	7	Male	Negative	Trauma	−	−
3	10	Female	Negative	Pneumonia	−	−
4	1	Male	Methomyl	Intoxication	−	−
5	1	Female	Negative	Respiratory failure	−	−
6	Adult	Male	Negative	DIC, gastric dilation	+	+
7	Adult	Male	Bromadiolone	Intoxication	+	+
8	Adult	Female	Strychnine	Intoxication	−	−
9	Adult	Female	Negative	Haemangiosarcoma	−	−
10	2	Female	Negative	Lymphoma	−	−
11	12	Male	Brodifacum	Intoxication	+	+
12	Adult	Female	Bromadiolone	Intoxication	+	+
13	5	Male	Bromadiolone	Intoxication	−	+
14	1	Female	Bromadiolone	Intoxication	+	+
15	Adult	Male	Coumatetralyl	Intoxication	+	+
16	13	Female	Brodifacum and bromadiolone	Intoxication	+	+
17	Adult	Female	Negative	Cardiac tamponade	−	−
18	Adult	Female	Brodifacum and bromadiolone	Intoxication	+	+
19	Adult	Male	Brodifacum and bromadiolone	Intoxication	+	+
20	Adult	Male	Coumatetralyl and brodifacum	Intoxication	+	+
21	Adult	Female	Negative	Renal Failure	−	−
22	Adult	Female	Negative	Hepatic Failure	+	−
23	Adult	Male	Negative	Haemangiosarcoma	−	−
24	Adult	Female	Negative	DIC, septic shock	+	+
25	Adult	Male	Embutramide	Intoxication	−	−
26	2	Male	Negative	Renal failure	−	−
27	Adult	Female	Negative	Cardiac failure	−	−
28	Adult	Male	Alphachloralose	Intoxication	−	−
29	1	Male	Negative	Hypovolemic shock	−	−
30	Adult	Female	Negative	Squamous cell carcinoma	−	−
31	Adult	Female	Brodifacum	Intoxication	+	+
32	Adult	Male	Negative	DIC due to massive Strongylosis	+	+
33	Adult	Female	Negative	Shock during surgery	−	−
34	Adult	Female	Negative	DIC, septic shock	+	+
35	Adult	Male	Negative	DIC, septic shock	+	+
36	Adult	Male	Negative	Respiratory failure	−	−
37	Adult	Female	Brodifacum	Intoxication	+	+
38	Adult	Female	Brodifacum	Intoxication	+	+
39	Adult	Female	Brodifacum and bromadiolone	Intoxication	+	+
40	Adult	Female	Negative	Hepatic failure	+	−
41	Adult	Male	Negative	Hepatic failure	+	−
42	Adult	Female	Negative	Cardiac failure	−	−
43	Adult	Male	Negative	DIC, septic shock	+	+
44	Adult	Female	Negative	Renal failure	−	−
45	Adult	Female	Negative	DIC, septic shock	+	+
46	6	Female	Negative	Lymphoma	−	−
47	Adult	Male	Negative	Hepatic failure with haemorrages	+	−
48	Adult	Female	Brodifacum	Intoxication	+	+
49	1	Male	Negative	Haemangiosarcoma	−	−
50	1	Male	Negative	Parvovirus infection	−	−
51	Adult	Female	Negative	Pulmonary thrombosis	−	−
52	Adult	Female	Negative	DIC, septic shock	+	+
53	Adult	Male	Negative	Haemangiosarcoma	−	−
54	Adult	Male	Negative	Haemangiosarcoma	−	−
55	Adult	Female	Negative	Haemangiosarcoma	−	−
56	Adult	Female	Negative	Haemangiosarcoma	−	−
57	Adult	Male	Negative	Haemangiosarcoma	−	−
58	Adult	Female	Negative	Haemangiosarcoma	−	−
59	Adult	Female	Negative	Haemangiosarcoma	−	−
60	Adult	Male	Negative	Haemangiosarcoma	−	−
61	Adult	Female	Negative	Haemangiosarcoma	−	−

**Table 2:** Toxicological results and description of the type of substance identified in the 20 dogs that had positive results for the toxicological analyses.

Case n.	Toxicology	Type of Substance
1	Brodifacum	Anticoagulant
4	Methomyl	Neurotoxic Insecticide
7	Bromadiolone	Anticoagulant
8	Strychnine	Neurotoxic
11	Brodifacum	Anticoagulant
12	Bromadiolone	Anticoagulant
13	Bromadiolone	Anticoagulant
14	Bromadiolone	Anticoagulant
15	Coumatetralyl	Anticoagulant
16	Brodifacum and bromadiolone	Anticoagulant
18	Brodifacum and bromadiolone	Anticoagulant
19	Brodifacum and bromadiolone	Anticoagulant
20	Coumatetralyl and brodifacum	Anticoagulant
25	Embutramide	Opioid
28	Alphachloralose	Narcotic
31	Brodifacum	Anticoagulant
37	Brodifacum	Anticoagulant
38	Brodifacum	Anticoagulant
39	Brodifacum and bromadiolone	Anticoagulant
48	Brodifacum	Anticoagulant

**Table 3:** Correlation between PIVKA-II immunohistochemistry on the liver and kidneys, as well as toxicological results.

	PIVKA-II n. Positive Cases	
	Liver	Kidney
Anticoagulant +	15/16	16/16
Anticoagulant −	12/45	8/45
Total	27/61	24/61

## Supplementary Materials

The following are available online at https://www.mdpi.com/article/10.3390/ani11092612/s1, Figure S1: Western blot analysis with antibody against PIVKA-II. A specific band of about 48–50 kDa is present for both protein lisate of liver and kidney. In the bottom lane vinculin as protein control.
