# Supplementary figures and images for: Abnormal Prothrombin (PIVKA-II) Expression in Canine Tissues as an Indicator of Anticoagulant Poisoning
Source: Animals (Basel). 2021 Sep 6;11(9):2612. doi: 10.3390/ani11092612 (PMC8466612; doi:10.3390/ani11092612)

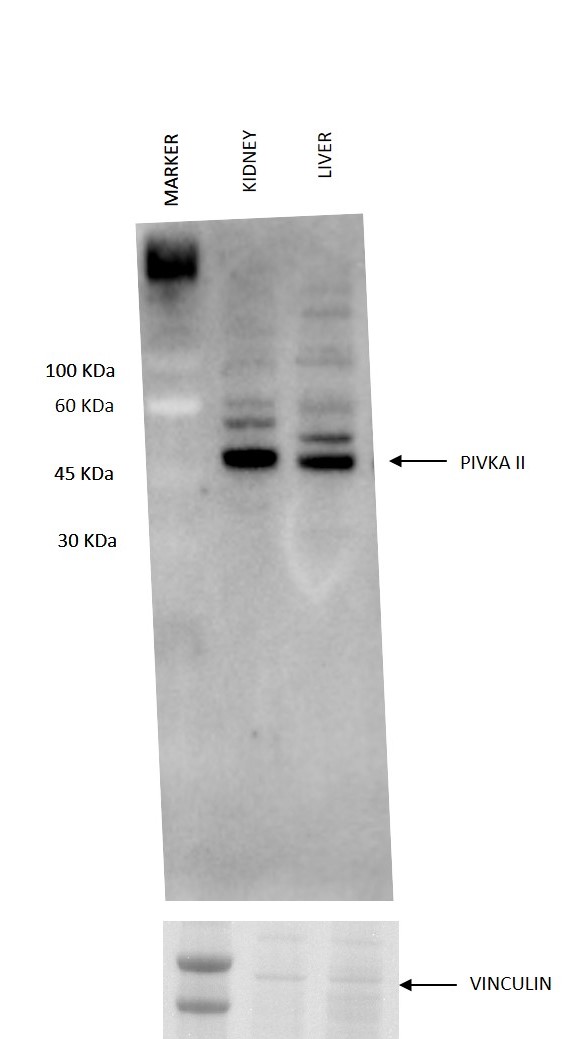

Supplement: Supplementary file 1 [file animals-11-02612-s001.zip › s1.jpg]
